# Meningitis and spondylodiscitis due to *Nocardia nova* in an immunocompetent patient

**DOI:** 10.1186/s12879-023-08067-5

**Published:** 2023-02-23

**Authors:** F. Lestin-Bernstein, M. Tietke, S. Schmiedel, M. Dreimann, O. Heese

**Affiliations:** 1 Clinical Hygiene and Infectiology, Helios Clinics of Schwerin - University Campus of Medical School Hamburg (MSH - University of Applied Sciences and Medical University), Wismarsche Str. 393-397, 19049 Schwerin, Germany; 2 Radiology and Neuroradiology, Helios Clinics of Schwerin - University Campus of Medical School Hamburg (MSH - University of Applied Sciences and Medical University), Schwerin, Germany; 3 Infectiology and Tropical Medicine, University Hospital Hamburg-Eppendorf, Hamburg, Germany; 4 Trauma Surgery and Orthopaedics, University Hospital Hamburg-Eppendorf, Hamburg, Germany; 5 Neurosurgery and Spinal Surgery, Helios Clinics of Schwerin - University Campus of Medical School Hamburg (MSH - University of Applied Sciences and Medical University), Schwerin, Germany

**Keywords:** *Nocardia nova*, Meningitis, Spondylodiscitis, Bacteraemia, Immunocompetent, Case report

## Abstract

**Background:**

Disseminated nocardiosis is a very rare disease. By now only few cases of meningitis and spondylodiscitis have been reported. To our knowledge, this is the first case of meningitis caused by *Nocardia nova*.

**Case presentation:**

We report on a case of bacteraemia, meningitis and spondylodiscitis caused by *N. nova* in an immunocompetent patient. We describe the long, difficult path to diagnosis, which took two months, including all diagnostic pitfalls. After nocardiosis was diagnosed, intravenous antibiotic therapy with ceftriaxone, later switched to imipenem/cilastatin and amikacin, led to rapid clinical improvement. Intravenous therapy was followed by oral consolidation with co-trimoxazole for 9 months without any relapse within 4 years.

**Conclusions:**

Establishing a diagnosis of nocardiosis is a precondition for successful antibiotic therapy. This requires close communication between clinicians and laboratory staff about the suspicion of nocardiosis, than leading to prolonged cultures and specific laboratory methods, e.g. identification by 16S rDNA PCR.

## Background

*Nocardia* are known to be low-pathogenic aerobic actinomycetes that are common in the environment and rarely cause disease, but when they do this occurs primarily in immunosuppressed individuals. *Nocardia nova* after inhalation of dust containing pathogens can lead to pulmonary and, less commonly, disseminated infections, usually in the form of brain abscesses. Prolonged antibiotic administration over 6–12  months is a prerequisite for successful therapy. Diagnosis is often delayed because the culturing of slow-growing bacteria is often unsuccessful under standard conditions. To our knowledge, this is the first description of meningitis caused by *Nocardia nova.*

## Case presentation

In March 2018, a 52-year-old Caucasian man presented at Schwerin hospital emergency room with headache that had been increasing for three days (left frontal region, pain scale 9 of 10), as well as nausea and photosensitivity, but no fever.

Lumbar spinal canal stenosis was diagnosed one year prior. In addition to conservative therapy, periradicular infiltrations were repeated for pain relief, most recently in an outpatient setting one month before admission. The patient, working in the field of landscaping, had no known previous diseases apart from one episode of acute schistosomiasis and dengue fever after a visit to Africa six years earlier and in particular no immunosuppressive diseases or therapies.

On admission, the patient was in severe pain (back pain and headache pain scale 9 of 10). Apart from meningism and lumbar pain on percussion, there were no further internal or neurological abnormalities. The cranial CT scan was normal, as were systemic inflammation parameters (CRP 6.3 mg/L [norm < 5 mg/L], no leucocytosis).

Due to a rising body temperature (38.1 °C) and the urgent suspicion of meningitis, a cerebrospinal fluid (CSF) puncture was done. Upon macroscopic examination, the CSF was slightly cloudy; accompanied by a mild leucocytosis of 251 × 10^6^/L (normal: < 5; differential blood count: 60% lymphocytes, 38% neutrophil granulocytes, 1% monocytes, 1 lymphoid cell) with significant lactate increase to 4.2 mmol/L, pronounced barrier disturbance (CSF/serum albumin ratio of 226; normal: < 8.0) and intrathecal immunoglobulin synthesis. No pathogen was detected initially (Gram and Auramine screening stain negative, no growth in conventional bacterial cultures after 7 days of incubation and in mycobacterial cultures until discharge, HSV and VZV PCR negative, syphilis serology negative). The cerebral MRI showed normal brain structures.

Empiric meningitis therapy was started with ceftriaxone 2 × 2 g daily (initially combined with ampicillin 6 × 2 g and acyclovir 3 × 700 mg daily, which were stopped after negative detection of *Listeria* spp. and HSV). The headaches subsided with this and the patient’s fever abated. In the follow-up CSF puncture after 7 days of ceftriaxone therapy, the lactate decreased slightly to 3.1 mmol/L. However, CSF pleocytosis persisted at 464 × 10^6^/L (86% lymphocytes, 8% neutrophil granulocytes, 4% lymphoid cells, 2% monocytes), as well as the pronounced barrier disorder with a CSF/serum albumin ratio of 114 and a three-class immunoglobulin reaction. CSF was positive in the tuberculosis liquid culture 6 days later and showed growth of dark-yellow colonies after 15 days of incubation on the tuberculosis solid culture media. This was assessed as contamination due to a negative *M. tuberculosis* complex PCR (including negative Auramine screening stain).

**Fig. 1:**
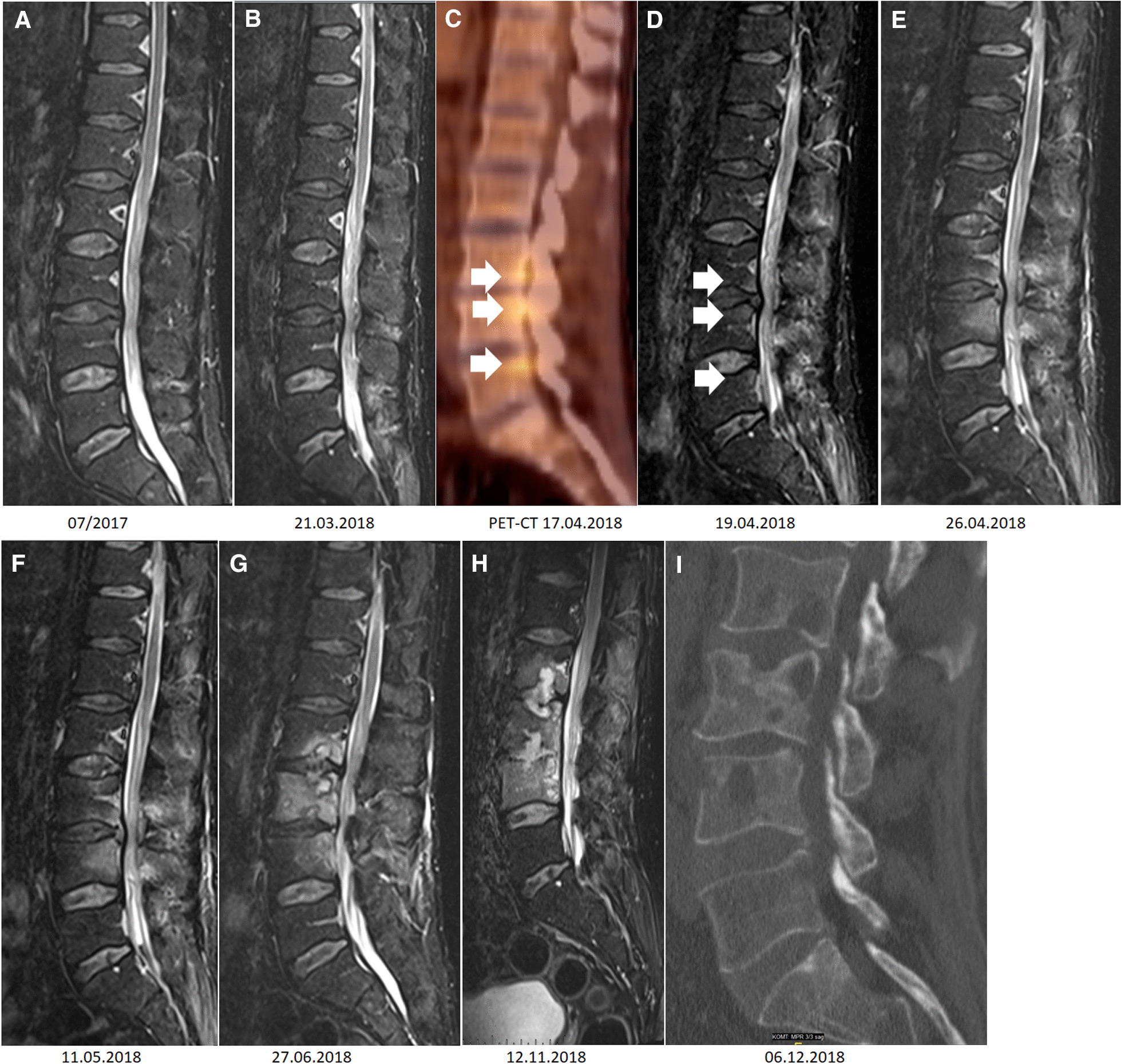
**a–i** MRI over the course and PET/CT. **a** Unremarkable sagittal STIR sequence of the lumbar spine from July 2017 with former radicular symptoms. **b** Multisegmental degenerative changes to the lumbar spine with progressive disc herniation at the level of L3/4. **c** Fluorine-18-fluorodeoxyglucose positron emission tomography (18F-FDG PET/CT) with hot spots with increased glucose activity dorsally on inferior endplate L3 and dorsally on superior endplates L4 and 5 (short white arrows). **d** Sagittal STIR sequence of the lumbar spine. Oedema of the endplates consistent with the PET/CT from **c** (short white arrows). **e**–**h**. Sagittal STIR sequence of the lumbar spine. Progressive oedema of L2-4 and regression of oedema in L5. Increasing destruction of the endplates within the context of spondylodiscitis. **i** Sagittal reformatted CT showing partial sclerosis of the vertebral bodies L2–L4. Furthermore, sharply demarcated lytic areas mainly associated with the endplates

Exacerbations of the known, chronic, recurrent sciatica symptoms occurred repeatedly during the inpatient stay. An MRI of the lumbar spine (see Fig. 1 b) showed multisegmental degenerative changes to the lumbar spine with progressive herniated disc at the level of L3/4 with emphasis in the right paramedian region and severe spinal narrowing with coiling of the caudal fibres at this level as the cause of the radicular symptoms. However, initially this was not interpreted as spondylodiscitis. After 14 days of ceftriaxone therapy, the patient was discharged in improved general condition.

**Fig. 2:**
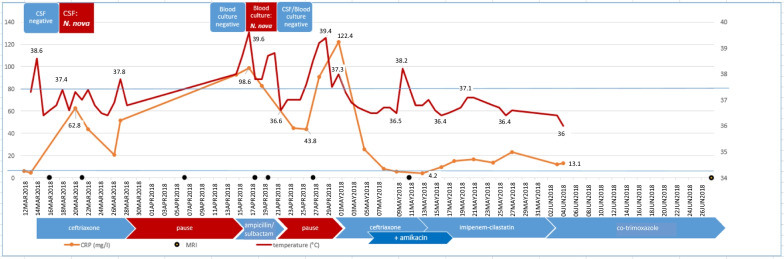
Clinical course: temperature, CRP, imaging and antibiotic history

In April 2018, one month after the initial visit (14 days after the end of ceftriaxone therapy; see also antibiotic history Fig. 2), nightly febrile episodes of up to 39 °C occurred again with chills, night sweats with laundry changes and progressive headache while lying down. Within one month, the patient had lost 5 kg in weight. At the time of rehospitalisation, the internal and neurological status was unremarkable except for the known radicular symptoms. There was no meningism, so another CSF puncture was primarily foregone. The laboratory tests on admission revealed leucocytosis of 12.4 × 10^9^/L and an elevated CRP of 93 mg/L with normal procalcitonin (0.1 mg/L). Due to a history of repeated stays in Africa, most recently in 2016, malaria was ruled out. The dengue, hepatitis, HIV, syphilis and rheumatoid serology as well as the QuantiFERON test were negative. There was no clear clinical improvement during calculated therapy with intravenous ampicillin/sulbactam. The patient continued to have a fever up to 39.6 °C, so antibiotic therapy was discontinued after 6 days in the absence of relevant pathogen detection.

The search for a focus initially did not reveal any ground-breaking findings: the abdominal ultrasound and whole-body CT scan were unremarkable. Echocardiography revealed no evidence of endocarditis. In the PET/CT performed 3 days after the repeat admission, increased focal radionuclide uptake was noted in the region of the small vertebral joints, the escaping spinal nerves and the adjacent vertebral bodies (see Fig. 1c), so the MRI of the lumbar spine was repeated four weeks after the one performed during the previous stay. In the examination without contrast medium, for the first time bony oedemas suggested haematogenous spondylitis, while repetition with contrast (see Fig. 1d, e), revealed progressive oedema and contrast enhancements with evidence of small abscesses in the autochthonous back muscles.

In the blood cultures taken on readmission, Gram-positive rods were grown after 5 days of incubation in one of four bottles, which could be identified as *Nocardia nova* using MALDI-TOF but were initially considered to be contamination from the environment. The other eleven blood culture bottles collected over the following 12 days remained negative (7 days of incubation).

CSF puncture was repeated 6 days after the end of antibiotic therapy due to an increase in fever up to 39.2 °C: cell count at 464 × 10^6^/L (61% neutrophil granulocytes, 2% lymphoid cells, 35% lymphocytes, 2% monocytes), lactate at 3.1 mmol/L still elevated, marked barrier disturbance of 282 and intrathecal immunoglobulin production. Due to the possible infectious aetiology, ceftriaxone 2 × 2 g daily was started again on the same day (see Fig. 2), with which the patient’s fever abated over the subsequent 3 days.

**Fig. 3:**
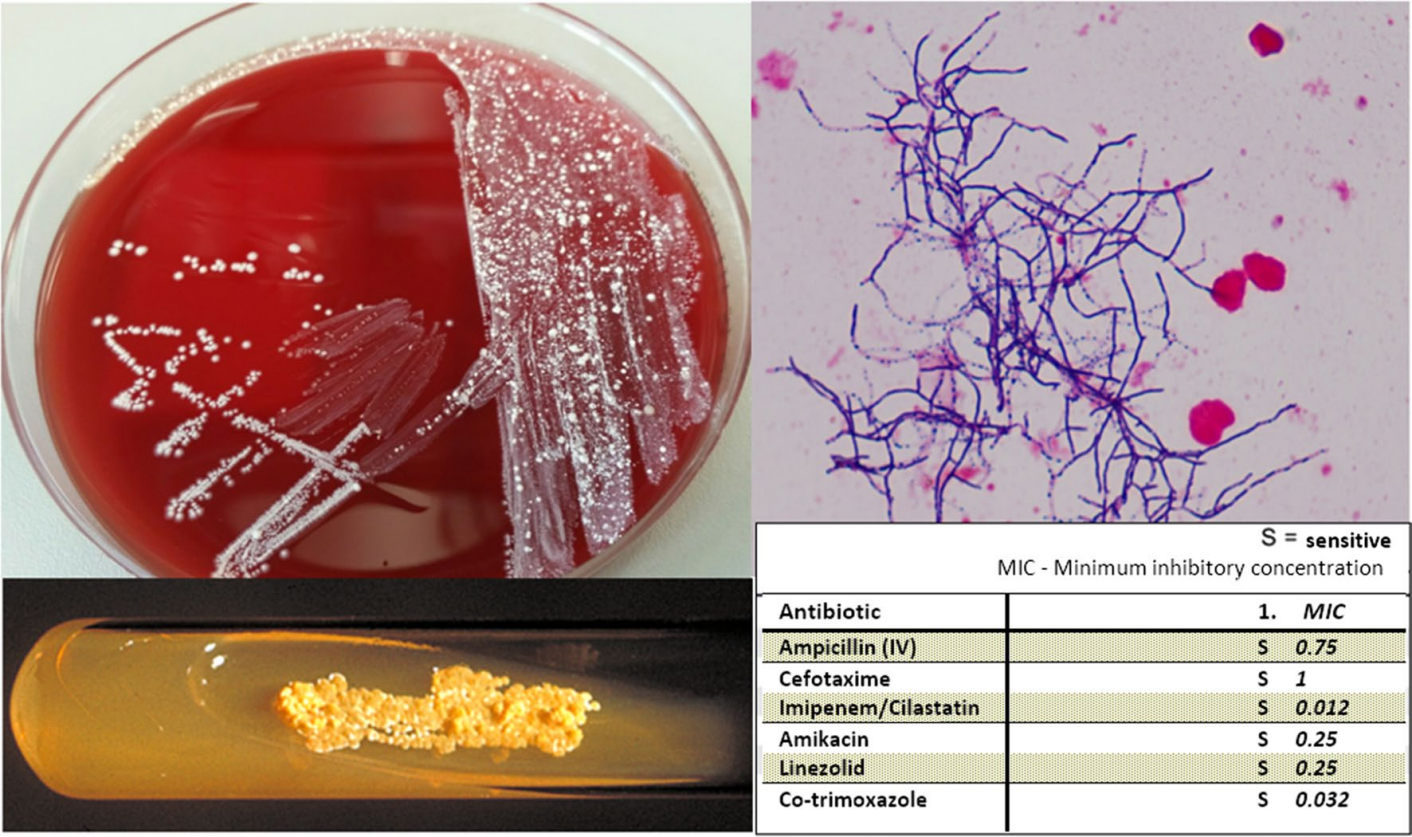
**A** *Nocardia nova *Growth on Columbia blood agar after 5 days of aerobic incubation and on tuberculosis culture media. **B** *Nocardia nova* (Gram-positive branched rods), Gram stain, 100 × and resistance testing

On 02 May 2018, after convening an interdisciplinary case conference (antimicrobial stewardship, internal medicine, microbiology, neurosurgery, neurology, neuroradiology) the “contamination” of the cerebrospinal fluid (dated 21 March 2018! during the first stay in hospital) was further differentiated as *Nocardia nova* using MALDI-TOF (matrix-assisted laser desorption/ionisation time-of-flight mass spectrometry) and parallel bacterial 16S rDNA-PCR with subsequent sequencing. More than seven weeks after the first visit to the emergency room, meningitis and spondylitis due to *Nocardia nova* were favoured as the main cause of the symptoms (see Fig. 3). Remarkably, *Noccardia* ssp. had been detected on both, standard media and mycobacterial cultures.

According to the general resistance situation of *Nocardia nova* [1], ceftriaxone was combined with amikacin 7.5 mg/kg once daily (see Fig. 2). Once resistance testing was present (in the absence of EUCAST limits, it was performed according to the CLSI standard M24-A2), ceftriaxone was replaced by imipenem/cilastatin 4 × 0.5/0.5 g after 14 days of therapy due to the lower minimal inhibitory concentrations (MIC) of imipenem/cilastatin (0.012 mg/L) compared to cefotaxime (1 mg/L; tested representative for ceftriaxone). Combination therapy with amikacin was continued for up to 14 days. There was a significant drop in inflammatory parameters (CRP of a maximum of 122 mg/L at the start of therapy within the normal range of < 5 mg/L 14 days later) (see Fig. 2). The headache stopped. The patient remained fever-free and with stable circulation throughout the rest of the hospital stay. A follow-up CSF puncture was not performed at the patient’s request. Persistent radicular pain symptoms could not be clearly differentiated from the pre-existing degenerative changes. No predisposing underlying disease could be determined (no evidence of a tumour disease despite comprehensive imaging and laboratory analysis, immunodeficiency screening negative, no diabetes mellitus) nor pulmonary involvement (chest CT, PET/CT and BAL negative). After a total of four weeks of intravenous therapy, co-trimoxazole (MIC 0.032 mg/L) was administered orally at an increased dose of 3 × 1,920 mg regarding the severity of the disease and the patient was discharged home. Due to initial gastrointestinal complaints, the dose was reduced to 3 × 960 mg 2 weeks later. Due to CSF space and bone involvement, we recommended continuation of therapy for at least around 12 months, but it was discontinued by the patient after 9 months. In the follow-up MRI after two months of therapy, the soft tissue oedema had already subsided, while the osseous structure reaction, as is known with other forms of osteomyelitis, lagged behind the clinical course, see Fig. 1g. The CRP remained within the normal range and the patient did not experience any new episodes of fever or headaches until now. However, after 6 months of therapy, he suffered from severe back pain partly due to a psoas abscess and to instability of the spine corresponding to massive bony lesions (see Fig. 1h, i). The abscess was drained without any pathogen detection by culture or molecular techniques. Since he refused surgical stabilisation, he was treated by physical therapy in combination with pain relief.

## Discussion

Nocardia is a genus of aerobic, branching, Gram-positive, partially acid-fast rod bacteria that occur ubiquitously in the soil and on plants and are not particularly virulent. Rarely, they can cause chronic granulomatous infections, either pulmonary due to inhalation of pathogen-containing dust or cutaneous/soft tissue infections from direct inoculation via the skin. In about half of cases, there is dissemination with spreading, particularly into the CNS. This is usually as a brain abscess. About two-thirds of cases involve immunosuppressed individuals, for example due to a tumour disease, HIV infection, diabetes mellitus or immunosuppressive therapies [2, 3].

*Nocardia nova* was first described and differentiated from the *Nocardia asteroides* complex as an independent species in 1983 mainly due to its individual resistance profile [4–6]. Species belonging to the *Nocardia nova* complex (*N. nova, N. elegans, N. veterana, N. kruczakiae, N. africana*) are generally sensitive to ampicillin (but not amoxicillin/clavulanic acid [2, 4]) and erythromycin, in addition to imipenem, amikacin, and co-trimoxazole, to which most *Nocardia* species are sensitive. The literature primarily describes pulmonary infections caused by *Nocardia nova*, followed by disseminated infections, usually involving the CNS in the form of brain abscesses. To our knowledge, isolated meningitis without brain abscesses (there was no evidence of this for our patient both clinically and on MRI) caused by *N. nova* has not been described, especially in an immunocompetent patient. Recently, Meena et al*.* [7] published a a systematic review of 206 reported cases of CNS nocardiosis of the last 20 years. They found isolated meningitis without brain abscess in only 5.8% of all patients, usually caused by *N. farcinica* [8–10]*.* CNS nocardiosis predominantly occurred in immunosuppressed individuals. Similar to our case, mild (usually < 1000 × 10^6^/L), predominantly granulocytic CSF pleocytosis with elevated lactate as an indication of bacterial infection was typical of nocardial meningitis.

*Spondylodiscitis* due to Nocardia is a rarity [11–13] and there are only three cases, *N. nova* has previously been reported as the cause of spondylodiscitis: in 2007, Hamdad described a female patient who was immunosuppressed due to a kidney transplant [14]. Pulmonary nocardiosis was known to be the primary focus for that patient and was clearly treated too briefly for three months. After one year of therapy (initially with imipenem/cilastatin, amikacin and co-trimoxazole, swapped to oral amoxicillin and erythromycin), the nocardial spondylodiscitis was resolved. The second report of spondylodisctis by *N.nova* by Zheng et al*.* focussed on surgical procedures to establish the diagnosis and disclaimed to describe underlying diseases and overall course [15]. The third case was published lately by Check et al*.*: a 54-year old female with *N. nova* spondylodiscitis and a history of untreated rheumatoid arthritis [16]. Apart from chronic pain, she resolved after 12 month of co-trimoxazole therapy.

In contrast, there was no evidence of immunosuppression or -deficit in our patient (extensive tumour screening, HIV, diabetes mellitus and rheumatic disease negative; no immunosuppressive therapies). The primary focus of the infection could not be conclusively clarified. For the patient working in the field of landscape gardening (and also in Africa), a healed pulmonary infection cannot be ruled out. The spine could represent the locus minoris resistentiae with dissemination due to the degenerative changes. However, a prior pulmonary infection could not be proven: neither in the patient’s history (cough or sputum in the past were denied) nor were there any corresponding residues in the chest CT. Alternatively, direct inoculation of the ubiquitous pathogens would be possible, for example during the PRT performed the previous year, most recently about one month before the start of the complaint. Isolated iatrogenic or postinterventional nocardial infections are described in the literature, but primarily in immunosuppressed patients [2, 17].

Nocardial infections may be underdiagnosed overall because evidence of slow-growing bacteria in culturing often exceeds the microbiological “standard methods” with 2–5 days of incubation. It is essential to convey the suspected diagnosis of “nocardiosis” and agree on extended incubation times of 10–14 (-28) days for corresponding material [2, 18] or, in rare cases, targeted direct pathogen detection by PCR [19]. Repeated collection of several relevant samples increases the probability of detection [12]. In our patient, *N. nova* was only able to be grown from one of three CSF punctures and one of twelve blood culture bottles. In the CSF, detection was only possible in the additional mycobacterial culture after 6 and 15 days (primarily assessed as contamination due to negative auramine stain *and tuberculosis complex* PCR). Only after parallel detection in the blood culture and an interdisciplinary case conference had taken place were the cultured pathogens identified as *Nocardia nova*. The procedure for the identification of unknown pathogen cultures from primarily sterile materials in our laboratory was subsequently changed: each pathogen cultured is differentiated to the species level, if possible. The species diagnosis of *Nocardia nova* was performed using MALDI-TOF MS, a method currently considered “state of the art” for timely routine diagnostics, confirmed by the more time-consuming 16S rDNA PCR with subsequent sequencing (“gold standard”). The strain corresponded to the expected resistance profile of the *N. nova* complex [1, 3, 4, 20] described in the literature.

The diagnostic challenge of the overall very rare disease was also reflected in the patient’s long ordeal with almost two months from initial presentation to the definitive diagnosis. If late diagnosis delays the start of therapy, there can be unfavourable, sometimes fatal outcomes [12].

Identification of the pathogen, is essential for successful therapy, since eradication of these slow growing bacteria requires *targeted therapy of at least six months* and for severe courses, immunosuppressed individuals or CNS or bone involvement, up to 12 months [2]. Typically, therapy contains *co-trimoxazole*, while initially, in severe cases, intravenous combination therapy of imipenem/cilastatin or ceftriaxone with amikacin should be more effective for 4–6 weeks [2, 11], for which larger studies on optimal antibiotic therapy and duration are lacking. In our patient, after 10 months of therapy, the infection healed without further complications apart from massive bony lesions. Even after a 4-year follow-up, there had been no relapse.

## Conclusions

Overall, disseminated nocardiosis, and nocardial meningitis and spondylodiscitis in particular, is a very rare disease. Because slow-growing *Nocardia* species are not usually grown under standard culture conditions, communication between the laboratory and clinic is crucial. Prolonged incubation (especially for invasively obtained, relevant materials) or in individual cases, direct diagnostics by molecular techniques should be performed. Interdisciplinary case conferences are also useful for this in order to avoid misinterpretation as “contaminants”.

## Data Availability

Not applicable.

## Abbreviations

*N. nova*: *Nocardia nova*
PCR: Polymerase chain reaction
CT: Computed tomography
CSV: Cerebrospinal fluid
HSV: Herpes simplex virus
VZV: Varicella zoster virus
MRI: Magnetic resonance imaging
PET: Positron emission tomography
MALDI-TOF: Matrix-assisted laser desorption/ionisation time-of-flight mass spectrometry
CRP: C-reactive protein
BAL: Bronchoalveolar lavage
MIC: Minimal inhibitory concentrations
EUCAST: European Committee on Antimicrobial Susceptibility Testing
